# Ovothiol Isolated from Sea Urchin Oocytes Induces Autophagy in the Hep-G2 Cell Line

**DOI:** 10.3390/md12074069

**Published:** 2014-07-07

**Authors:** Gian Luigi Russo, Maria Russo, Immacolata Castellano, Alessandra Napolitano, Anna Palumbo

**Affiliations:** 1 Institute of Food Sciences, National Research Council, Avellino 83100, Italy; E-Mail: mrusso@isa.cnr.it; 2 Laboratory of Cellular and Developmental Biology, Stazione Zoologica Anton Dohrn, Naples 80121, Italy; E-Mail: immacolata.castellano@szn.it; 3 Department of Chemical Sciences, University of Naples Federico II, Naples 80126, Italy; E-Mail: alesnapo@unina.it

**Keywords:** ovothiols, autophagy, sea urchin, Hep-G2

## Abstract

Ovothiols are histidine-derived thiols isolated from sea urchin eggs, where they play a key role in the protection of cells toward the oxidative burst associated with fertilization by controlling the cellular redox balance and recycling oxidized glutathione. In this study, we show that treatment of a human liver carcinoma cell line, Hep-G2, with ovothiol A, isolated from *Paracentrotus lividus* oocytes, results in a decrease of cell proliferation in a dose-dependent manner. The activation of an autophagic process is revealed by phase contrast and fluorescence microscopy, together with the expression of the specific autophagic molecular markers, LC3 II and Beclin-1. The effect of ovothiol is not due to its antioxidant capacity or to hydrogen peroxide generation. The concentration of ovothiol A in the culture media, as monitored by HPLC analysis, decreased by about 24% within 30 min from treatment. The proliferation of normal human embryonic lung cells is not affected by ovothiol A. These results hint at ovothiol as a promising bioactive molecule from marine organisms able to inhibit cell proliferation in cancer cells.

## 1. Introduction

The marine environment is characterized by a high biodiversity of species, which accounts for the enormous chemical diversity representing a great potential source of bioactive molecules. This has led to the discovery of several hundreds of novel compounds, whose biological properties and biotechnological applications are being intensively investigated [1,2].

Among these, a new class of sulfur-containing amino acids, thiohistidine derivatives, termed ovothiols, have been isolated as disulfides in three different forms, A, B and C, differing in the degree of methylation. In particular, ovothiol A is unmethylated, whereas ovothiol B and C are mono- or di-methylated at the aminoacidic amino group, respectively. These metabolites are present in ovary, eggs and biological fluids of various marine invertebrates and some fishes [3,4,5,6,7,8,9]. Ovothiols have also been found in human pathogens, such as *Leishmania major* and *Trypanosoma cruzi* [10,11], and in some microalgae [12]. 

Recently, a renewed interest in ovothiols has been raised from the identification and characterization of a 5-histidylcysteine sulfoxide synthase (OvoA), the enzyme that catalyzes the first step of their biosynthesis [13,14,15]. *In silico* analysis of homologous OvoA enzymes revealed that they are encoded in more than 80 genomes from proteobacteria to animalia. 

The wide occurrence of ovothiols in various organisms points to their involvement in different biological processes. Indeed, ovothiols have been reported to play a key role in sea urchin, since they protect the embryo from the high oxidative burst at fertilization, reacting with hydrogen peroxide with a rate constant five times greater than glutathione [6,7]. Moreover, it has been suggested that ovothiols are involved in the protection of some pathogens from oxidative stress during infection [16] and in the regulation of the redox control of chloroplasts [12]. *In vitro* studies revealed that ovothiols are potent antioxidants; they react with a variety of radicals with efficiency comparable to that of ascorbic acid and the tocopherol analogue, trolox [17]. Starting from ovothiols, many derivatives have been synthesized and their antioxidant properties examined in *in vitro* systems [18,19,20,21]. One of these compounds has been shown to be a potent agent in mammalian cerebroprotection [22]. Further biological activities have been poorly investigated. 

**Figure 1:**
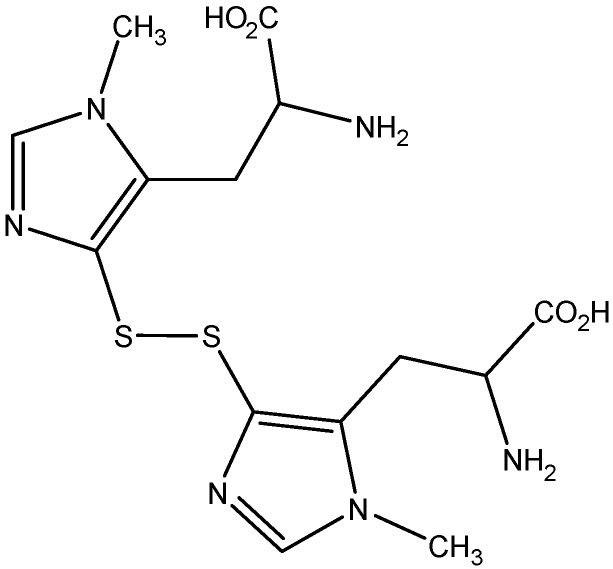
Structure of ovothiol A disulfide.

In the present study, the biological activity of ovothiol A disulfide (Figure 1) purified from sea urchin *Paracentrotus lividus* eggs has been tested on a human liver carcinoma cell line, Hep-G2. Treatment with increasing concentrations of ovothiol A resulted in a decrease of cell viability with a concomitant occurrence of autophagy, as assessed by fluorescence microscopy and the expression of specific autophagic molecular markers.

## 2. Results

### 2.1. Isolation of Ovothiol A

**Figure 2:**
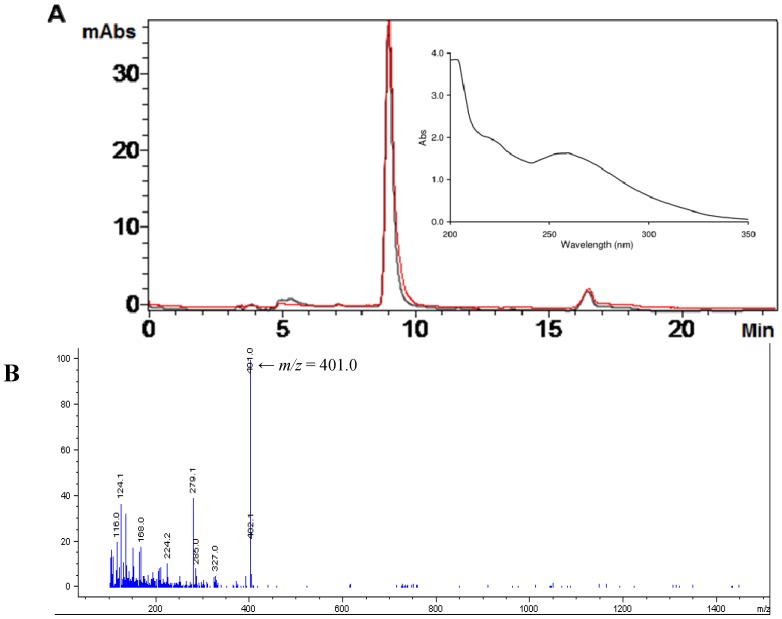
Analysis of ovothiol A purified from sea urchins. (**A**) Elutographic profile of ovothiol A obtained by ion exchange chromatography purification of the sea urchin extracts. Detection at 254 (black trace) and 280 (red trace) nm. Inset: UV-Vis absorption spectrum of ovothiol A; (**B**) Mass spectrum of ovothiol A.

Ovothiol A was isolated from eggs of the sea urchin, *Paracentrotus lividus*, by the procedure previously developed with some modifications [4]. The homogenate was freed from protein by treatment with acidic ethanol overnight and from the lipid component by ethyl ether extraction and then fractionated by ion exchange chromatography with HCl gradient elution. The 4 M HCl eluates showing a broad absorption centered at 260 nm were neutralized to pH 8 and allowed to stand in air to get the conversion of any ovothiol A into the disulfide. Further purification by ion exchange chromatography with 2 M HCl elution afforded pure ovothiol A disulfide (reverse phase HPLC analysis, detection at 254 and 280 nm), identified by the comparison of the elutographic, absorption and mass properties (RT 9 min, eluant A, A280/A254 = 1, ESI (+) MS *m/z* 401 [M + H]^+^) (Figure 2) with those of an authentic sample, previously isolated from sea urchin oocytes and characterized by ^1^H-NMR and ^13^C-NMR spectra (see the Experimental Section for ^1^H-NMR and ^13^C-NMR data) [3,4]. 

### 2.2. Anti-Proliferative Effects of Ovothiol A in the Hep-G2 Cell Line

**Figure 3:**
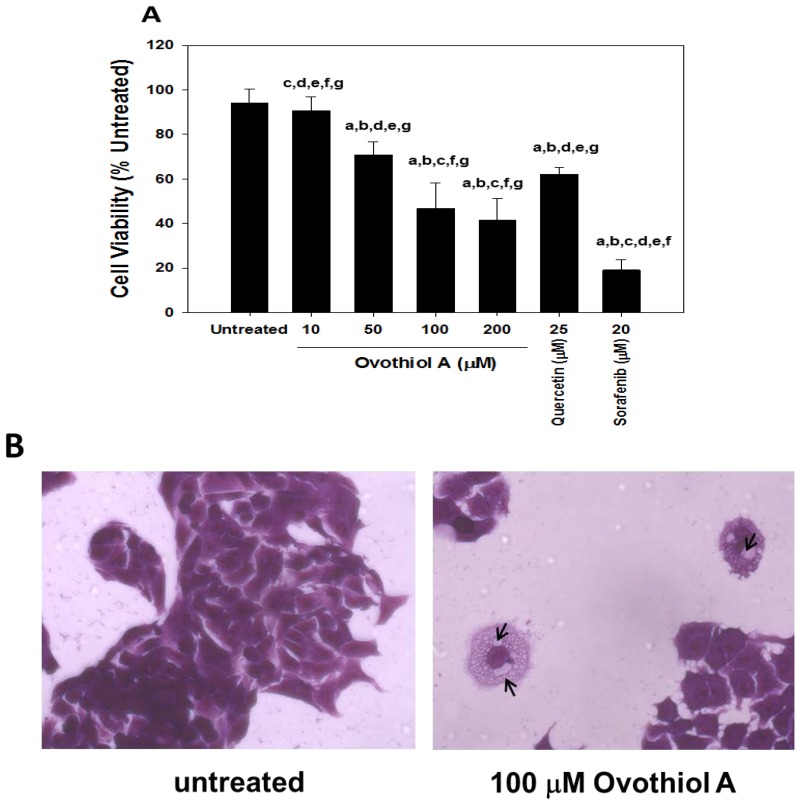
Ovothiol A induces a dose-dependent cytotoxicity in Hep-G2 cells. (**A**) Cells were treated for 24 h with increasing doses of ovothiol (10–200 μM) or positive controls (quercetin and sorafenib at 25 μM and 20 μM, respectively), and cell viability was measured by the crystal violet assay. Values are presented as the mean ± SD compared to untreated cells. Symbols (a, b, c, d, e, f, g) indicate significance: *p* < 0.001 with respect to untreated (a) and treated cells (b = ovothiol 10 μM; c = ovothiol 50 μM; d = ovothiol 100 μM; e = ovothiol 200 μM; f = quercetin 25 μM, g = sorafenib 20 μM) (one-way ANOVA test); (**B**) Representative images of cells treated with ovothiol A at 100 μM (optical microscope Axiovert 200 M Zeiss; 400×, bright field). The arrows indicate the presence of vacuoles in treated cells.

To assess whether ovothiol A was able to interfere with cell proliferation, Hep-G2 cells were incubated in the presence of different concentrations of ovothiol A for 24 h. The crystal violet dye assay was employed to measure the viability and proliferation of cells after incubation. Ovothiol A was cytotoxic in a dose-dependent manner with a maximum effect in the range of 50–100 μM (Figure 3A). At 24 h, the decrease in cell viability was of 24% and 52% at 50 and 100 μM, respectively, compared to untreated controls. Similar effects were obtained on the treatment of Hep-G2 cells with comparable concentrations of ovothiol C, isolated from *Sphaerechinus granularis* eggs [4] (data not shown). 

A representative picture of the effects of ovothiol A on Hep-G2 proliferation is shown in the micrographs reported in Figure 3B. The limited number of dead cells with the concomitant presence of vacuoles and an altered cell morphology was suggestive of the activation of an autophagic process. Quercetin and sorafenib, whose capacity to induce autophagy has been previously documented, were employed as positive controls (Figure 3A,B) [23,24].

### 2.3. Sea Urchin Ovothiol A Activates Autophagic Processes in the Hep-G2 Cell Line

**Figure 4:**
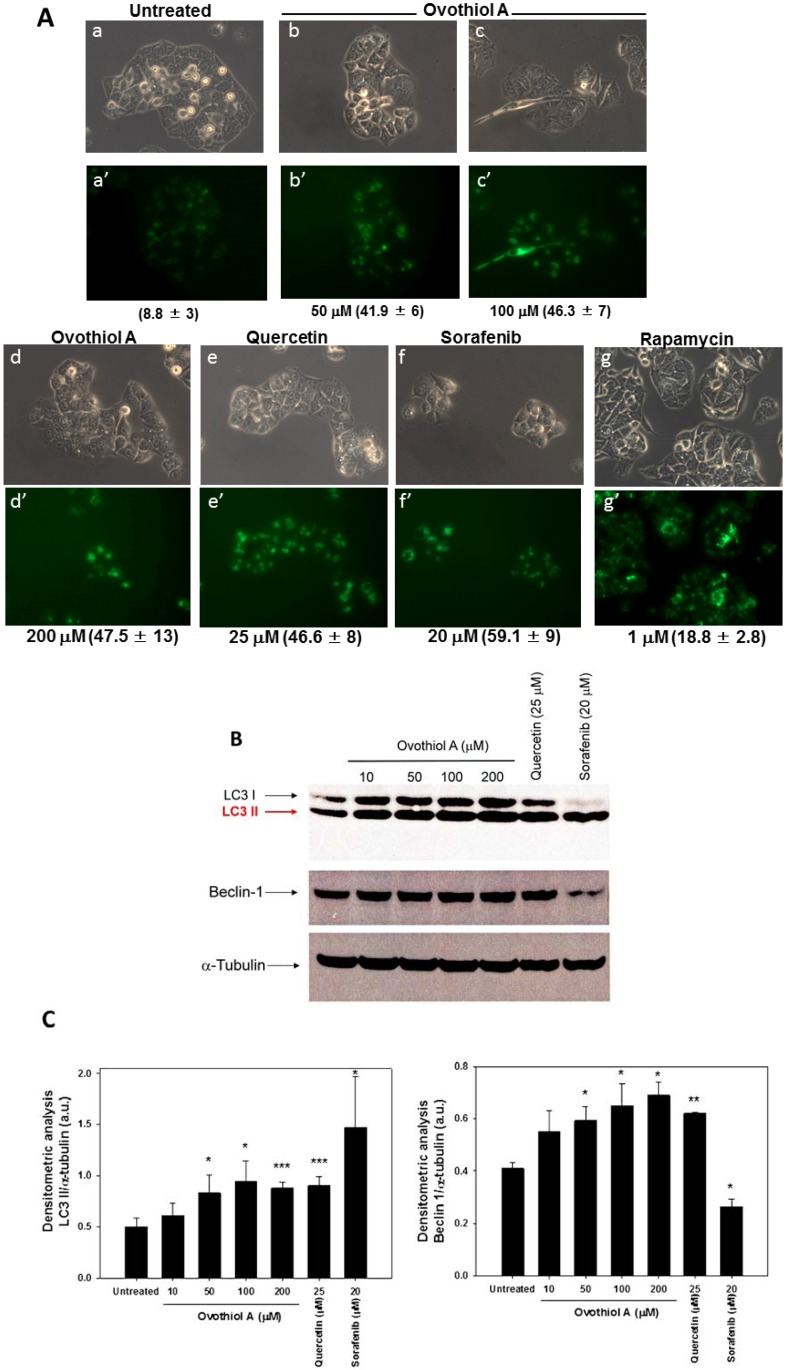
Ovothiol A induces autophagy in Hep-G2 cells. (**A**) After stimulation for 24 h with increasing doses of ovothiol (50–200 μM) or positive controls (quercetin, sorafenib and rapamycin, at 25, 20 and 1 μM, respectively), autophagy was detected and measured using a specific kit, as described in the Materials and Methods Section. At least 100 cells in two independent fields were counted using a phase contrast microscope, and the presence of positive autophagic cells was evaluated by fluorescence in the same fields. The figure shows representative images of cells treated with ovothiol, quercetin, sorafenib and rapamycin at the indicated concentrations (optical microscope Axiovert 200 M Zeiss; 400×, phase contrast in **a**–**g** and fluorescence FITC in **a’**–**g’**). Numbers on the bottom of panels **a**’–**g**’ indicate percentage (means ± SD compared to untreated cells of two separate experiments) of positive autophagic cells; (**B**) Western blot analysis of LC3 I, LC3 II and Beclin-1 expression in Hep-G2 cells treated for 24 h with indicated doses of ovothiol, quercetin and sorafenib. Bands are representative of one out of three separate experiments performed; (**C**) Densitometric analysis of blots shown in Panel B (optical density of LC3 II/α-tubulin, left and Beclin-1/α-tubulin, right). Values are presented as the mean ± SD Symbols indicate significance: *p* < 0.05 (*), *p* < 0.01 (**), *p* < 0.001 (***) with respect to untreated cells.

The presence of vacuoles within Hep-G2 cells treated with ovothiol A (Figure 3B) suggested the activation of an autophagic process. To verify this hypothesis, we used multiple assays to detect autophagy and to avoid false-positive results [25]. Hep-G2 cells were stained with Cell-ID™ Green autophagy dye (Vinci-Biochem, Vinci, FI, Italy), a fluorescent reagent able to specifically incorporate autolysosomes [23]. Immunofluorescence staining indicated the presence of autolysosomal vacuoles in cells incubated with ovothiol A (Figure 4A), with a maximum effect in the range of 100–200 μM (46%–47%). As positive controls, we treated Hep-G2 cells for 24 h with 25 μM quercetin, 20 μM sorafenib and 1 μM rapamycin, (Figure 4A, panels e,f,g and e’,f’,g’). The vacuoles stained by Cell-ID™ Green were clearly visible under phase contrast microscopy (Figure 4A, top panels). The result of the Cell-ID™ Green assay overlapped with the quantification obtained by measuring the fluorescence emitted by vacuoles (FITC) and by normalizing with that deriving from nuclei (Hoechst, Vinci-Biochem, Vinci, FI, Italy) through a spectrofluorimetric reading (data not shown).

The activation of an autophagic process was confirmed by immunoblots, which showed the increased expression of microtubule-associated protein 1A/1B-light chain 3 (LC3) II and Beclin-1 bands after 24 h of ovothiol A incubation (Figure 4B). Densitometric analyses of the relevant bands are reported in Figure 4C. The former is a lipidated form of the soluble LC3 I and is a factor essential for autophagosome formation [26]; the latter, Beclin-1, is the mammalian orthologue of yeast Atg6, which interacts with several cofactors to form core complexes, thereby inducing autophagy [27]. 

**Figure 5:**
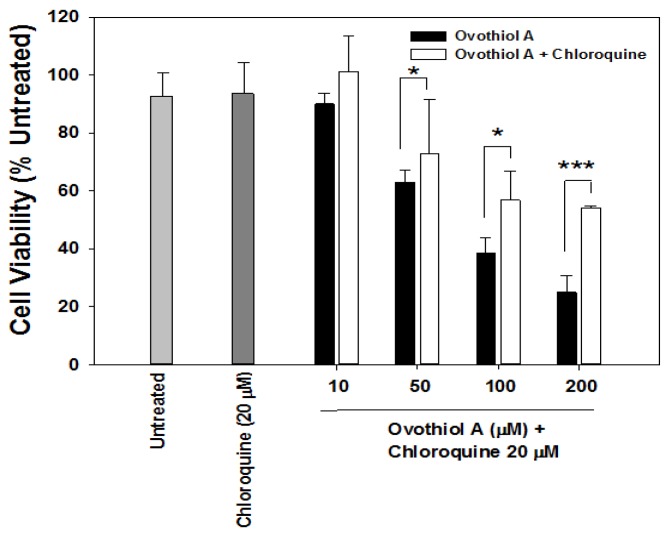
Ovothiol A induces autophagy-dependent cell death in Hep-G2 cells. Cells were pre-treated with 20 μM chloroquine for 1 h followed by the addition of the indicated doses of ovothiol A (10–200 μM) for 24 h. Cell viability was measured by the crystal violet assay. Symbols indicate significance: *p* < 0.05 (*) and *p* < 0.005 (***) between ovothiol A and chloroquine plus ovothiol co-treated samples.

To assess whether the cytotoxicity measured in Figure 3A was a consequence of the autophagic process triggered by ovothiol A, we employed chloroquine, a compound largely accepted in the literature as an autophagy inhibitor [28,29,30,31]. As reported in Figure 5, the co-treatment with chloroquine for 24 h protected Hep-G2 cells from ovothiol-induced cell death at the concentrations at which ovothiol was effective. 

### 2.4. Ovothiol A Recovery in the Culture Media of the Hep-G2 Cell Line

**Figure 6:**
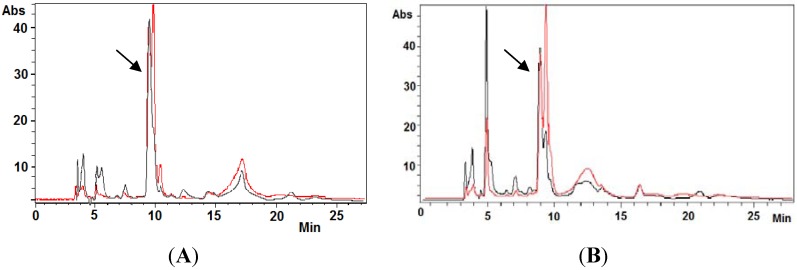
Ovothiol A levels decrease in the incubation medium. The HPLC trace of the Hep-G2 culture medium following treatment with 200 μM ovothiol; (**A**) T = 0 min; (**B**) T = 30 min. Detection at 254 (black trace) and 280 (red trace) nm. Highlighted is the peak corresponding to ovothiol A.

Hep-G2 cell cultures treated with 200 μM ovothiol A were examined for the presence of ovothiol A, compared to untreated control cells. At different times, culture media and cellular pellet extracts were examined by HPLC for the presence of ovothiol A. After 10 and 30 min of incubation, ovothiol A in culture media was reduced to 80% and 76% compared to 0 min (Figure 6). 

The analysis of cell lysates even after 30 min of incubation failed to reveal the presence of appreciable (detection limit < 10 μM) amounts of ovothiol A, nor of its reduced thiol form. The possibility that the loss of ovothiol A was due to sequestration into the cell membrane was ruled out by solubilization of the cellular debris after the removal of the lysates with SDS, followed by HPLC analysis, which, however, did not show appreciable amounts of the compound.

### 2.5. ROS Production in the Hep-G2 Cell Line

**Figure 7:**
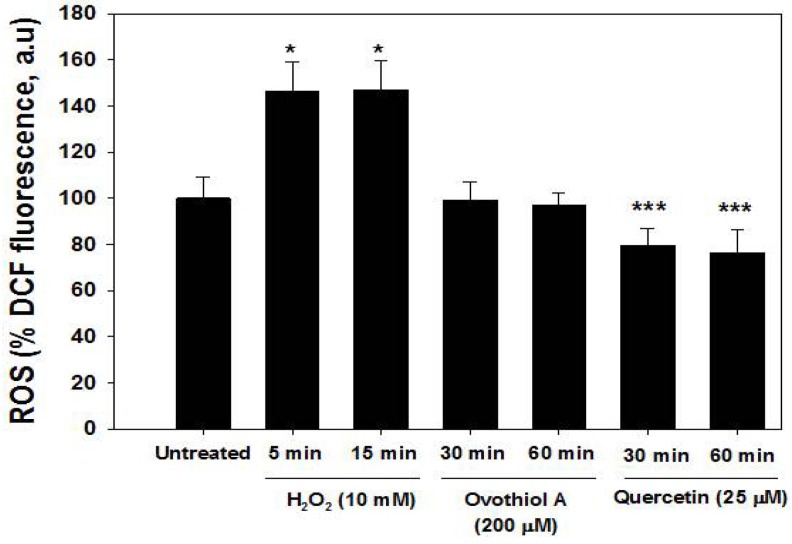
Ovothiol A does not decrease ROS levels in Hep-G2 cells. Cells were treated for 30 and 60 min with the maximal dose of ovothiol (200 μM), 25 μM quercetin and H_2_O_2_ 10 mM (5 and 15 min positive control). ROS levels were measured by the DCF assay as described in the Materials and Methods Section. Bar graphs represent the mean (expressed as the percent of untreated) ± SD. Symbols indicate significance: *p* < 0.001 (***); (*) *p* < 0.05 with respect to untreated samples.

To assess whether the effects induced by ovothiol A were related to its antioxidant capacity, the level of ROS in Hep-G2, after treatment with ovothiol A at the highest concentration employed in previous experiments, was measured. As reported in Figure 7, when cells were incubated with the ROS indicator, DCFH-DA, no significant fluorescence decrease was observed compared to untreated cells, ruling out any antioxidant action of ovothiol A. As controls, we employed H_2_O_2_ (10 mM) and quercetin (25 μM) as oxidant and antioxidant agents, respectively, to demonstrate that Hep-G2 can sense different ROS concentrations.

Similarly, we excluded the possibility that the cytotoxicity associated with the treatment with ovothiol A could derive from the generation of H_2_O_2_ following the interaction between ovothiol A and cell culture medium components, an artifact previously described for several naturally occurring molecules [32,33]. By means of the ferrous oxidation-xylenol orange (FOX) assay (Experimental Section), the formation of H_2_O_2_ was measured after the incubation of 200 μM ovothiol A in culture medium in the absence of cells. No detectable amounts of H_2_O_2_ were produced after 30–60 min (data not shown).

### 2.6. Ovothiol A Is Not Cytotoxic on Immortalized Fibroblast

WI-38 are diploid human embryonic lung cells often used as an example of non-malignant cells with respect to the cancer cell line [34,35]. In fact, WI-38 are diploid cells with a normal karyotype, a finite lifetime and a cell division cycle that is normally regulated. Since we tested the effect of ovothiol A on a cell line where cell division is dysregulated, we selected WI-38 as a possible normal counterpart of Hep-G2 cells.

**Figure 8:**
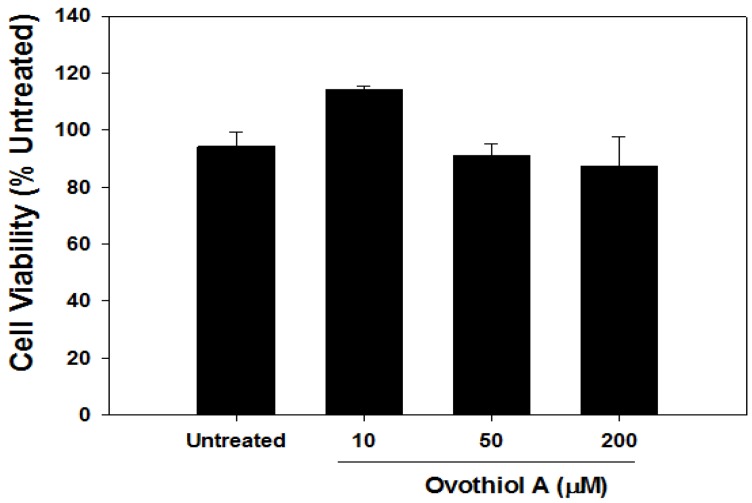
Ovothiol does not induce cytotoxicity in WI-38 cells. Cells were treated for 24–48 h with increasing doses of ovothiol A (10–200 μM), and cell viability was measured by the crystal violet assay. Values are presented as the mean ± SD compared to untreated cells.

When incubated in the presence of increasing concentrations of ovothiol A, no significant cytotoxicity (<10%) was observed, even at the highest concentration tested (Figure 8). Paradoxically, treatment with 10 μM ovothiol A slightly, but not significantly, increased cell proliferation, suggesting the activation of a hormetic process frequently occurring when drugs and natural compounds are tested at low concentrations [36,37]. This possibility may be explored in the near future.

## 3. Experimental Section

### 3.1. Animals

Sea urchins were collected during the breeding season in the Gulf of Naples from a location that it is not privately-owned nor protected in any way, according to the authorization of Marina Mercantile (Italian Republic presidential decree 1639/68, 09/19/1980 confirmed on 01/10/2000). The field studies did not involve endangered or protected species. All animal procedures were in compliance with the guidelines of the European Union (Directive 609/86). The animals were transported in an insulated box to the laboratory within 1 h after collection and maintained in tanks with circulating sea water until testing. To induce gamete ejection, sea urchins were injected with a KCl 0.5 M solution through the peribuccal membrane. Eggs were washed with sea water and filtered through gauze to remove pieces of spicules. The collected eggs were centrifuged at 2000× *g* for 10 min, and the precipitate containing the eggs was kept at −20 °C until use.

### 3.2. Chemicals and Reagents

Crystal violet, formalin, acetic acid, dimethylsulfoxide (DMSO), chloroquine and Dowex^®^ 50WX2, 200–400 mesh, were purchased from Sigma-Aldrich (Milan, Italy); H_2_O_2_ was from Carlo Erba Reagents (Milan, Italy); rapamycin was from Enzo Life Science, PBS (phosphate-buffered saline) tablets and DCFH-DA (dichlorofluorescein-diacetate) were purchased from Life Technologies (Monza, Italy).

### 3.3. Isolation of Ovothiol A Disulfide

Sea urchin eggs (10 g) were homogenized in ethanol–1 M HCl 80:20 *v/v* (65 mL) and left overnight at room temperature under stirring in the air. After centrifugation at 14,000× *g* for 15 min at 4 °C, the supernatant was recovered. The pellet was washed three times with acidic ethanol, and the combined supernatants, concentrated to a small volume, were extracted three times with ethyl ether (50 mL) freed from peroxide by passage over an alumina column. The aqueous layer was taken to a small volume and loaded onto a Dowex 50WX2, column (1 cm × 22 cm). Elution was sequentially carried out with water, 0.1 M, 0.5 M HCl. The column was then eluted with 4 M HCl, and the collected fractions were monitored spectrophotometrically in the 200–350 nm range. Fractions exhibiting the UV spectrum typical of ovothiol (see Figure 2) were collected, concentrated to a small volume and oxidized in the presence of air, for 4 h at pH 8. After acidification to pH 2, the sample was re-chromatographed on the same Dowex column. Fractions exhibiting the UV spectrum of ovothiol were collected and taken to dryness, affording a colorless, glassy solid (2.5 mg). The purity of the compound was checked by LC-MS analysis run on LC/MSD Agilent 1100 VL: R_T_ 9 min, 1% formic acid taken to pH 4.5, with ammonia as the eluent (Eluent A), *m*/*z* 401 ([M + H]^+^). ^1^H (^13^C) spectra were run at 400 (100.1) MHz on a Bruker Instrument. ^1^H-NMR spectrum (D_2_O): δ 3.44 (2H × 2, d, *J* = 7.5 Hz, βCH_2_), 3.94 (3H × 2, s, N-Me), 4.29 (1H × 2, t, *J* = 7.5 Hz, αCH), 8.95 (1H × 2, s, H-2). ^13^C-NMR spectrum (D_2_O): δ 25.5 (CH_2_), 34.0 (CH_3_), 54.5 (CH), 130.0 (C), 134.8 (C), 141.1 (CH), 173.3 (C).

### 3.4. Cell Culture and Viability

The Hep-G2 cell line, derived from a human hepatocellular carcinoma [38], and WI-38 from ATCC [39] were maintained in Dulbecco’s modified Eagle’s medium (DMEM) supplemented with 10% fetal bovine serum (FBS; Lonza, Belgium), 1% l-glutamine, 1% penicillin, 1% streptomycin (Life Technologies) at 37 °C, in a 5% CO_2_ humidified atmosphere and harvested at approximately 90% confluence. For viability experiments, cells were plated in a 48-multiwell plate at a density of 3–5 × 10^4^/mL in a total volume of 0.5 mL and allowed to adhere for 24 h. Subsequently, cells were treated for 24 h with ovothiol A at the concentrations indicated.

Cell viability was determined by the crystal violet assay [40]. Briefly, medium was carefully removed and the cells gently washed with PBS. After fixing with 10% formalin for 15 min to room temperature, 0.1% crystal violet (*w/v*) was added and the cells incubated at room temperature for 30 min. Prior to the dye solubilization, cells were photographed in bright field (magnification 400×) using an inverted microscope (Axiovert 200 Ziess, Jena, Germany). After washing, cells were added with 10% acetic acid to solubilize the dye, and the absorbance was measured spectrophotometrically at 590 nm. Experiments have been done in triplicate and were repeated 3 times.

### 3.5. Measurement of Autophagy

Autophagic cell death was monitored by using the Cell-ID™ Autophagy Detection Kit (Vinci-Biochem, Vinci, FI, Italy) using quercetin and sorafenib as the positive control [23,24]. The Cell-ID™ Green autophagy dye is used as a selective marker of autolysosomes and earlier autophagic compartments. One day prior to staining, cells (3 × 10^4^) were seeded in a 24-multiwell plate and allowed to grow for 24 h under cell culture conditions. After incubation with ovothiol A (10–200 μM) for 24 h, Hep-G2 cells were washed with assay buffer and incubated with the autophagy detection marker diluted 1:500 in DMEM without phenol red (Life Technologies, Milan, Italy) supplemented with 5% FBS for 30 min. Cells were rinsed with assay buffer and observed by fluorescence microscopy (Zeiss Axiovert 200, Milan, Italy). Those observed in the same field were counted both in phase contrast and fluorescence to evaluate the presence of vacuoles. Experiments were performed twice in duplicate, and 100 cells per well were counted.

### 3.6. Immunoblots

After treatments, cells (0.5 × 10^6^) were resuspended in lysis buffer, as reported [29], and following the measurement of protein concentration, total protein lysates (20 μg) were loaded on a 4%–12% pre-cast gel (Novex Bis-Tris pre-cast gel 4%–12%; Life Technologies, Milano, Italy) using MES (2-(*N*-morpholino)ethanesulfonic acid) buffer, according to the manufacturer’s protocol. Immunoblots were performed following standard procedures and using as primary antibodies anti-LC3 (Cell Signalling; Milano, Italy), anti-Beclin-1 (GeneTex, Prodotti Gianni Milano, Italy) and anti-α-tubulin (Sigma-Aldrich, Milan, Italy). PVDF membranes were finally incubated with horseradish peroxidase-linked secondary antibody against mouse or rabbit (GE Healthcare, Milano, Italy) and immunoblots developed using the ECL Plus Western Blotting Detection System Kit (GE Healthcare, Milan, Italy). Band intensities were quantified measuring optical density on a Gel Doc 2000 Apparatus (Bio-Rad Laboratories, Milan, Italy) and Multi-Analyst Software (Bio-Rad Laboratories, Milan, Italy).

### 3.7. Intracellular ROS Measurement

ROS production was assayed using 2′-7′-dichlorofluorescein diacetate (DCFH-DA; Life Technologies, Milan, Italy), a non-fluorescent product that freely permeates cells. Hep-G2 cells were stimulated 5 and 15 min with H_2_O_2_ 10 mM (positive control), 200 μM ovothiol A or 25 μM quercetin (30–60 min) and incubated at 37 °C in 5% CO_2_. After several washes with PBS, cells were incubated for 30 min with 10 μM DCFH-DA at 37 °C in 5% CO_2_. When DCFH-DA penetrates membrane, the diacetate group is hydrolysed by cellular esterase, and then, DCFH is oxidized to a fluorescent molecule, 2′-7′-dichlorofluorescin (DCF), by intracellular peroxides. After incubation, fluorescence was spectrofluorimetrically determined (FL-500; Bio-Tek Instruments, Milan, Italy) with an excitation setting of 485 ± 20 nm and an emission setting of 530 ± 20 nm. Experiments have been done in quadruplicates and repeated 2 times.

### 3.8. Ferrous Oxidation-Xylenol Orange (FOX) Assay

Cell culture medium (RPMI/10%FCS) was incubated for 30–60 min in the presence of PBS (control) or ovothiol A in a 96-well microtiter plate (total volume 0.2 mL). Subsequently, 0.1 mL of the incubated medium was mixed with 0.9 mL of FOX reagent (1:9 *v/v* of 1 mM xylenol orange, 2.5 mM Fe(NH_4_)_2_(SO_4_)_2_ and 4.4 mM 2,6-di-tertbutyl-4-methylphenol in methanol). After 30 min, samples were centrifuged in a microfuge at maximal speed for 5 min, and the absorbance of the supernatants was determined spectrophotometrically at 560 nm. Results were expressed as hydrogen peroxide equivalent [41]. Experiments have been done in triplicate and repeated 3 times.

### 3.9. Analysis of Ovothiol A in Cell Cultures

Hep-G2 cells (1.2 × 10^6^ cells) were cultured in the absence and presence of 200 μM ovothiol A and collected after incubation at different time intervals. The culture media are recovered by centrifugation and directly analyzed by HPLC for ovothiol A quantitation. The cellular pellets are suspended in PBS, sonicated and the supernatants examined by HPLC analysis. Experiments were run in duplicate. HPLC analyses were run on an LC10AD instrument equipped with binary pumps and a Shimadzu SPD-10AVP detector set at 254 nm and 280 nm. A Phenomenex Synergi Sphereclone octadecylsilane (25 cm × 0.46 cm, 4 μ particle size) column was used with Eluent A, at a 0.7 mL/min rate.

## 4. Discussion

The results of this study provide evidence that ovothiol A has an anti*-*proliferative effect on the human hepatocellular carcinoma Hep-G2 cell line, leading to the activation of autophagy, a complex and well-controlled catabolic process, which usually allows cells to survive stress signals, like nutrient deprivation, by recycling its own cytoplasmic components through lysosomal pathways [31]. This process, called “macroautophagy” or simply “autophagy”, is different from other forms of catabolic recycling, involving proteasome and chaperone pathways (microautophagy). Autophagy is not only a process allowing cells to resist metabolic stress, because, if cells do not recover this gap, they die with a form of programmed cell death (PCD Type 2), genetically and morphologically different from apoptosis (PCD Type 1) and necrosis (Type 3) [42,43]. The morphological changes that define autophagy include a slow formation of double-membrane-formed vacuoles, which incorporate parts of the cytoplasm subsequently digested by lysosomal hydrolases (reviewed in [31]). As a matter of fact, the presence of vacuoles within Hep-G2 cells after treatment with ovothiol A has suggested the occurrence of autophagy.

From a mechanistic point of view, autophagy includes several steps: initiation, vesicle nucleation, elongation process, docking and fusion, vesicle breakdown and degradation. The phagophore (also called the isolation membrane) sequesters material in double-membraned vesicles in autophagic vacuoles. Vesicle nucleation activates the mammalian PI_3_K multiprotein complex, which includes Beclin-1. The elongation process may involve the conjugation of phosphatidylethanolamine to LC3, leading to the conversion of the soluble form LC3 I to the lipidated form LC3 II, essential for autophagosome formation. Autophagosomes undergo maturation by fusion with lysosomes to create autolysosomes, where the inner membrane and the luminal content of the autophagic vacuoles are degraded by lysosomal enzymes (see [44] for a review). The fluorescence staining of autolysosomes, together with the increased expression of the two autophagic markers, LC3 II and Beclin-1, clearly demonstrate that ovothiol A induces these autophagic steps in Hep-G2 cells. Considering that the BH3 domain of Beclin-1 is bound to and inhibited by Bcl-2 or Bcl-XL, a functional relationship between autophagy and apoptosis could be envisaged. The use of chloroquine enforces this concept. This molecule is known as an antimalarial drug that inhibits lysosomal acidification and is used in autophagy-related studies as an inhibitor, since it impairs autophagosome-lysosome fusion and lysosomal degradative activity [31]. The presence of chloroquine abolished the cytotoxicity induced by ovothiol A, demonstrating that cell death is dependent on autophagy. Considering the relationships between autophagy and cancer [45], the ovothiol A-induced autophagy does not protect cell survival, but contributes to cell death, at least in the Hep-G2 cell line. 

Since autophagy is an intracellular lysosomal degradation process, induced under stress conditions, the role of ROS, generated from mitochondria or external sources, is usually assessed [46]. However, our data excluded that the effect of ovothiol A on Hep-G2 cells was due either to its antioxidant capacity or to H_2_O_2 _generation. 

Our HPLC data indicated that the concentration of ovothiol in the culture medium decreased by about 20% within 30 min, suggesting that the effective concentration of the compound is significantly lower than the administered concentration. On the other hand, the amounts found inside the cells were below the detection limits, and also, the possibility of sequestration into the cell wall was ruled out. It is possible, therefore, that the failure to detect ovothiol A inside the cells is due to its interaction with other cellular components, e.g., redox exchange with intracellular thiols, or with free cysteine residues of proteins, as demonstrated for glutathione [47,48]. The finding that both ovothiol A and ovothiol C have the same effect on Hep-G2 cells demonstrated that methylation did not affect the bioactivity of these compounds.

A possible weakness of this work may be represented by the relatively high concentrations of ovothiol A applied to Hep-G2 cells. However, two main considerations support the view of a significant effect of ovothiol A: (1) we demonstrated that “normal” cells, such as WI-38 (Figure 8), are resistant to the doses applied to Hep-G2 cells, suggesting that the biological effects exerted by ovothiol A on Hep-G2 cells are not due to non-specific cytotoxicity, but it is the result of the activation of well-defined cellular processes (autophagy); (2) preliminary data [49] on at least two additional cell lines showed that the cytotoxic effect of ovothiol A was measured at lower concentrations (10–25 μM) compared to Hep-G2. Future efforts will be devoted to ameliorate the pharmacological properties of ovothiol A. 

Other bioactive molecules, isolated from plants, animals, marine organisms and microorganisms, have been shown to induce autophagy [50,51,52]. Our results that ovothiol A induced autophagy of Hep-G2 cell line are relevant, considering the great interest in the connection between autophagy and cancer cell metabolism [53]. 

## 5. Conclusions

This study enforces the enormous importance of the marine environment as a source of potential pharmacological compounds. Indeed, we clearly demonstrated that ovothiol A, isolated from sea urchin eggs, induced the autophagy-dependent cell death of human hepatic cancer cells, being inactive on normal cells. Several pharmacological inhibitors and small interfering RNAs, able to affect key steps in the autophagic process, represent potential anticancer drugs. Future studies will be directed to investigate the potential therapeutic effects of ovothiol A. 

## Abbreviations

Ovo A: 5-histidylcysteine sulfoxide synthase
LC3: Microtubule-associated protein 1A/1B-light chain 3
